# Genomic Analysis Of NAD+ Synthesis Pathways Involved In Aging and Cancer

**DOI:** 10.1093/geroni/igab046.2510

**Published:** 2021-12-17

**Authors:** Jeremy Meyers, Raul Castro-Portuguez, Luis Espejo, George Sutphin

**Affiliations:** 1 University of Arizona, Tucson, Arizona, United States; 2 The University of Arizona, Tucson, Arizona, United States; 3 UNIVERSITY OF ARIZONA, TUCSON, Arizona, United States

## Abstract

Cancer cells have elevated energy demands to sustain continuous growth and other malignant processes and undergo extensive metabolic reprogramming to meet these demands. One element of this reprogramming in many cancer subtypes is elevated synthesis of nicotinamide adenine dinucleotide (NAD+), a critical co-enzyme that supports energy production through both glycolysis and the TCA cycle. The kynurenine metabolic pathway is the evolutionarily conserved means by which cells produce NAD+ de novo from tryptophan. NAD+ levels drop with age, a contributing factor to many forms of age-related disease. While interventions that increase NAD+ have been shown to extend lifespan, previous work from our lab demonstrates that knockdown of several kynurenine pathway enzymes, thus decreasing de novo NAD+ production, results in increased longevity of Caenorhabditis elegans by 20-30%. To address this apparent contradiction, we propose that kynurenine pathway inhibition may produce metabolic feedback that results in upregulation of NAD+ recycling. Eukaryotic cells recycle NAD+ from nicotinamide (NAM) through one of two pathways: the Salvage pathway in mammalian cells and the Preiss-Handler pathway in C. elegans and related invertebrates species. We are using tools in C. elegans and human cell culture to examine the interaction between kynurenine/de novo NAD+ synthesis and NAD+ recycling through Salvage and Preiss-Handler. In particular, we are interested in how combining interventions between these pathways will influence activity throughout the NAD+ metabolic networks (measured via mass spectrometry), physiological phenotypes, and transcriptomic changes (via RNA sequence data) involved in aging and age-associated disease.

